# A Nutraceutical Approach for Hypertension: Randomized Controlled Trial of Grape Pomace Extract and L-Arginine

**DOI:** 10.3390/antiox15030329

**Published:** 2026-03-05

**Authors:** Federico Abate, Elisabetta Schiano, Mariano Stornaiuolo, Fabrizia Guerra, Anna Terracciano, Gaetano Piccinocchi, Eugenio Caradonna, Fulvio Ferrara, Gian Carlo Tenore, Ettore Novellino

**Affiliations:** 1 Department of Environmental, Biological and Pharmaceutical Sciences and Technologies, University of Campania Luigi Vanvitelli, Via Vivaldi 43, 81100 Caserta, Italy; 2 NGN Healthcare—New Generation Nutraceuticals s.r.l., Torrette Via Nazionale 207, 83013 Mercogliano, Italy; 3 Department of Pharmacy, University of Naples Federico II, Via Domenico Montesano 59, 80131 Naples, Italy; 4 Comegen S.C.S., Società Cooperativa Sociale di Medici di Medicina Generale, Viale Maria Bakunin 41, 80125 Naples, Italy; 5 Integrated Laboratory Medicine Services, Centro Diagnostico Italiano S.p.A., 20011 Milan, Italy; 6 Department of Experimental and Clinical Pathology, IRCCS Istituto Auxologico Italiano, 20149 Milan, Italy; 7 Department of Medicine and Surgery, Catholic University of the Sacred Heart, 00168 Rome, Italy

**Keywords:** hypertension, polyphenols, L-arginine, nutraceutical, nitric oxide, blood pressure, clinical trial, oxidative stress

## Abstract

Hypertension remains a major global health challenge, and pharmacological therapy is often constrained by tolerability issues. Adjunctive approaches targeting the nitric oxide synthase and soluble guanylate cyclase–cyclic guanosine monophosphate (sGC–cGMP) pathway may offer additional benefits. This study investigated the efficacy and safety of a nutraceutical formulation combining grape pomace extract (Taurisolo^®^) and L-arginine in patients with grade 1 and grade 2 hypertension. The formulation was designed to enhance nitric oxide (NO) bioavailability and support sGC–cGMP signaling. Taurisolo^®^, a polyphenol-rich extract, is known for its antioxidant and endothelial-protective properties, while L-arginine serves as the physiological substrate for endothelial NO synthase. Clinical outcomes included blood pressure changes, renal function parameters, and health-related quality of life assessed through the SF-12 questionnaire. Supplementation with Taurisolo^®^ plus L-arginine resulted in significant and sustained reductions in systolic and diastolic blood pressure, with renal function remaining stable throughout the study. Participants also reported meaningful improvements in perceived health, emotional well-being, vitality, and social functioning. The intervention was well tolerated, with no major adverse effects. These findings support the potential of Taurisolo^®^ combined with L-arginine as a safe and effective adjunctive strategy to conventional antihypertensive therapy, warranting further mechanistic investigation.

## 1. Introduction

Hypertension is classified as stage 1 when the systolic pressure ranges from 130 to 139 mmHg or the diastolic pressure ranges from 80 to 89 mmHg. Stage 2 hypertension is characterized by a systolic pressure of ≥140 mmHg or a diastolic pressure of ≥90 mmHg [1]. This is based on evidence from numerous randomized studies showing that treating patients with these BP values leads to benefits in the prevention and management of systemic diseases [2,3]. Based on ambulatory measurement, the global prevalence of hypertension was estimated at 1.1 billion individuals in 2015, with an incidence of over 150 million in Central and Eastern Europe [2]. The continuous relationship between blood pressure and the risk of cardiovascular events has been demonstrated at all ages and across all ethnic groups and extends from high BP levels to relatively low values [4]. Hypertension becomes progressively more common with advancing age, with a prevalence above 60% in people over 60 years of age [5]. SBP becomes the dominant risk predictor with age, while diastolic elevations are more common in younger adults (<50 years) as DBP tends to change from middle age onward as a consequence of arterial stiffening [6]. Meta-analyses of randomized trials including several hundred thousand patients have shown that a 10 mmHg reduction in SBP or a 5 mmHg reduction in DBP is associated with a substantially greater than 20% reduction in all cardiovascular events, a 10–15% reduction in all-cause mortality, a 35% reduction in stroke, a 20% reduction in coronary events and a 40% reduction in heart failure [7]. As populations age, adopt more sedentary lifestyles and gain body weight, the global incidence of hypertension will continue to increase; this high prevalence of hypertension is consistent worldwide, regardless of income status [2]. Mortality rates alone do not provide a complete picture of the disease burden borne by people in different populations. Overall disease burden is assessed using disability-adjusted life years (DALYs), a time-based measure that combines years of life lost due to premature mortality (YLLs) and years lived in a disabled state due to permanent disability (YLDs). One DALY represents the loss of the equivalent of one year of full health [8]. Elevated blood pressure was the leading global risk factor contributing in 2015 to more than 200 million DALYs [9], an increase of 40% since 1990 [9]. A systolic pressure of at least 140 mmHg accounts for most of the mortality and DALY burden (70%) [9]. Hypertension has an independent and continuous relationship with the incidence of numerous cardiovascular events (hemorrhagic stroke, ischemic stroke, myocardial infarction, sudden death, heart failure and peripheral artery disease) [10]. Further, hypertension is the risk most related to developing atrial fibrillation (AF) [11], while emerging evidence links early increases in blood pressure to an increased risk of cognitive decline and dementia [12]. Factors that increase the risk of hypertension can be distinguished into modifiable risk factors, which include an unhealthy dietary pattern (excessive salt intake, diet high in saturated and trans fats, low intake of fruits and vegetables), physical inactivity, tobacco and alcohol use, overweight or obesity, and non-modifiable risk factors, which include a family history of hypertension, age over 65 years and comorbidities such as diabetes or kidney disease [13]. Treatment of the majority of patients with hypertension requires pharmacological therapy, in addition to lifestyle measures, to achieve optimal blood pressure control [1]; the main classes of drugs recommended for the treatment of hypertension are ACE inhibitors, angiotensin receptor blockers (ARBs), β-blockers, α-blockers, calcium channel blockers (CCBs) and thiazide and thiazide-like diuretics such as chlorthalidone and indapamide [1,13,14]. Although antihypertensive drugs are associated with a broad range of side-effect symptoms—such as dry cough with ACE inhibitors; dizziness with ARBs; rashes, constipation and peripheral edema with calcium channel blockers; bradycardia, sleep problems and gastrointestinal symptoms with beta blockers; and drowsiness, postural hypotension or dry mouth with alpha blockers—these adverse effects consistently emerge as one of the leading causes of poor adherence [15]. Together with patients’ doubts about the necessity of long-term treatment due to a limited perception of hypertension as a serious condition, concerns about medication dependence, the daily life burden of chronic therapy, and the fluctuating, often irregular dosing patterns typical of real-world behavior substantially undermine persistence with therapy [16,17]. For this reason, recent scientific research has focused on alternative therapeutic remedies that are safer, preferably of natural origin, which can promote a more physiological regulation of blood pressure and support classical pharmacological therapies in order to reduce drug doses and consequently side effects. Very recent clinical research has focused on the soluble guanylate cyclase (sGC)–cGMP pathway as an important therapeutic target and one of the main signaling cascades for the regulation of blood pressure whose impairment can lead to hypertension [18,19]. This signaling requires L-arginine supply, which is the substrate used by nitric oxide synthase (NOS) enzymes (endothelial eNOS, neuronal nNOS and inducible iNOS) to generate nitric oxide (NO) [20]. NO is an endogenous gaseous mediator that diffuses across cell membranes and binds cytosolic soluble guanylate cyclase (sGC), a heterodimeric protein composed of α and β subunits containing a heme-iron H-NOX domain that binds NO. Subsequently, NO binding to sGC triggers a conformational change and activates the catalytic site to convert GTP into the second messenger cGMP, which in turn binds and activates mainly protein kinases (PKG1 and PKG2) crucial for the regulation of vascular tone and thus blood pressure [21]. Pathophysiological impairment of NO–sGC–cGMP signaling contributes to blood pressure dysregulation [22]. Attenuation or interruption of this pathway may be caused by decreased bioavailability of endogenous L-arginine due to increased arginase activity, resulting in reduced NO production and consequently lower tissue cGMP levels that may be associated with the development of hypertension; therefore, dietary L-arginine supplementation as an NO source to restore cGMP-dependent signaling is a logical approach [23]. It is also important to emphasize that sGC activity is highly sensitive to cellular oxidative stress which can oxidize the heme iron from Fe^2+^ to Fe^3+^ and thereby reduce sGC responsiveness to NO and lower cGMP production, impairing the functional homeostasis of affected organs including blood pressure regulation [24]. Increased oxidative stress can also impair eNOS function, reducing endogenous NO production, or oxidize NO into peroxynitrite (NO_3_^−^), all of which contribute to reduced cGMP formation and related pathological consequences including hypertension [25].

Beyond traditional oxidative mechanisms, recent experimental evidence indicates that microbially derived metabolites can contribute to endothelial dysfunction [26]. In particular, trimethylamine N-oxide (TMAO) has been shown experimentally to inhibit eNOS competing with L-arginine on the biding site of the enzyme, hindering NO production and acetylcholine-mediated vasorelaxation in rat aortas, suggesting an additional pathway by which metabolic factors can reduce NO bioavailability and impair NO–sGC–cGMP signaling. Moreover, mass spectrometry analysis confirmed that TMAO prevents L-arginine’s conversion to L-citrulline, while not affecting smooth muscle responses, as SNP-induced relaxation in endothelium-denuded rings remained unchanged, confirming that the inhibitory effect was endothelium-dependent [27].

In this scenario, dietary supplementation with antioxidant molecules may be another useful approach to preserve physiological homeostasis [28]. Natural polyphenols may be particularly useful because, as hydrophilic molecules, they can diffuse into the cytosol and interact with catalytic sites of protein subunits, preventing oxidation of physiological metal species including iron ions [29]. Moreover, in vitro and ex vivo experiments showed that a polyphenol-rich grape pomace extract was particularly effective in counteracting the adverse cardiovascular effects caused by excess circulating TMAO [30]. A head-to-head comparison of grape pomace extract and L-arginine, administered individually versus in combination, was previously performed in hypertensive rodent models, showing that the combined treatment produced greater improvements in endothelial function and blood pressure-related endpoints than either component alone [27,31].

In light of the considerations outlined above, we decided to evaluate the effects of a combination of a polyphenol-rich grape pomace extract (Taurisolo^®^) [32] and L-arginine in hypertensive patients in order to provide a natural and safe alternative for BP regulation, potentially complementary to traditional pharmacological therapies. Given these observations, the primary objective of this study was to evaluate whether the treatment could effectively normalize or reduce SDP and DPB values. Also, secondary endpoints were included: markers of renal function and quality of life.

## 2. Materials and Methods

### 2.1. Study Design

The study was designed as a monocentric, randomized, double-blind, parallel-group clinical trial. Participants were identified through the patient registry of general practitioners affiliated with COMEGEN—Società Cooperativa Sociale (Naples, Italy). All documentation required for trial initiation—including the full study protocol, study synopsis, informed consent forms, patient information sheets, and the letter of intent—was submitted to the Campania Territorial Ethics Committee 1 (Comitato Etico Territoriale Campania 1) (Via Mariano Semmola 52, Naples, 80131, Italy) for review and approval. The study was approved by the committee (ref: n° 5/25 of 29 April 2025) and conducted in accordance with the Declaration of Helsinki of 1964 (as revised in 2000). This study is listed on the ISRCTN registry (www.isrctn.com) with ID ISRCTN99673579 (https://www.isrctn.com/ISRCTN99673579, accessed on 26 November 2025). Participants eligible for inclusion in the study were male and female individuals of Caucasian ethnicity, aged between 18 and 75 years. Eligible participants were Caucasian men and women aged 18–75 years, with a documented diagnosis of essential hypertension established at least 12 months prior to enrollment and under stable antihypertensive therapy (ACE inhibitors or ARBs, either as monotherapy or in combination with diuretics) that could not be modified during the study. Only individuals without additional cardiological (atrial fibrillation, heart failure) or metabolic (diabetes, dyslipidemia) comorbidities who were able to understand and provide written informed consent were considered. Exclusion criteria included autoimmune, rheumatologic, or vascular diseases other than essential hypertension; severe hypertension (grade 3, ≥171/106 mmHg); diabetes; irregular sleep–wake cycles (e.g., night-shift work within the preceding 3 months); hypercholesterolemia (>250 mg/dL) or hypertriglyceridemia (>200 mg/dL); recent cardiovascular events (myocardial infarction or stroke within 6 months); renal impairment (creatinine > 1.5 mg/dL) or hepatic dysfunction (ALT/AST or γ-GT > 2× upper normal limit); anemia (Hb < 12 g/dL) or other chronic diseases; habitual intense physical activity; gastrointestinal disorders; weight variation > 3 kg in the 3 months prior to enrollment; malignant neoplasms; significant neurological or psychiatric disorders, including alcohol or drug abuse; and concurrent therapy with hypoglycemic agents, laxatives, cyproheptadine, antidepressants, antiserotonergic drugs, phenothiazines, barbiturates, oral corticosteroids, or antipsychotics. Women who were pregnant, breastfeeding, or of childbearing potential without adequate contraception were also excluded.

**Figure 1:**
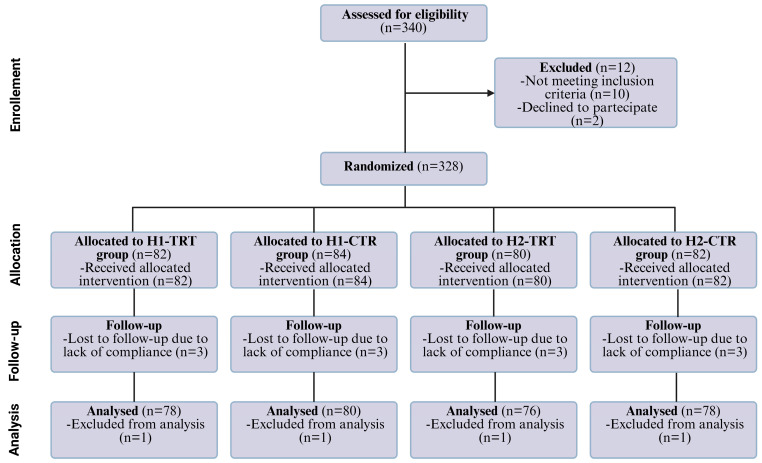
CONSORT flow diagram. Abbreviations: H1-TRT, grade 1 hypertension treated group; H1-CTR, grade 1 hypertension control group; H2-TRT, grade 2 hypertension treated group; H2-CTR, grade 2 hypertension control group. Created in BioRender. Abate, F. (2026) https://BioRender.com/2425zjb (accessed on 1 January 2026).

Figure 1 illustrates the participant flowchart, developed in accordance with CONSORT (Consolidated Standards of Reporting Trials) guidelines [33]. A total of 340 individuals were initially enrolled as potentially eligible. After the screening phase, 12 participants were excluded for not meeting the inclusion criteria, with 328 patients subsequently randomized to the allocated treatment groups. Of these, 312 participants completed the study, while the remaining individuals discontinued participation for reasons detailed in the flowchart.

**Figure 2:**
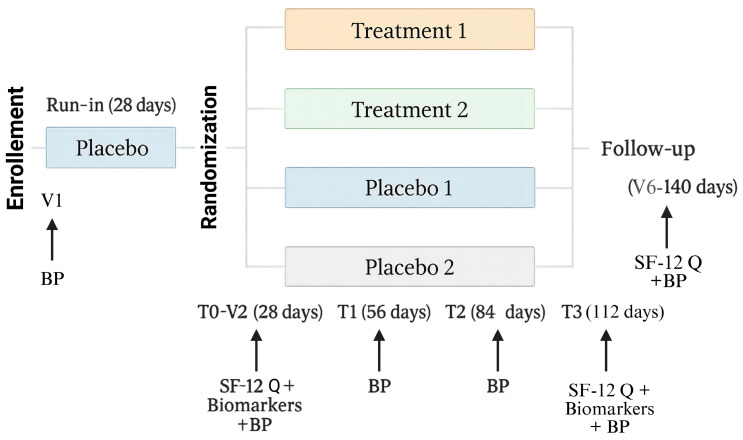
Flow-chart of study. Abbreviations: BP, Blood Pressure; SF-12 Q, Short Form Health Survey questionnaire.

Participants were randomized into four parallel groups according to hypertension grade (Figure 2). Treatment Group 1 included subjects with grade 1 hypertension (SBP 130–150 mmHg, DBP 90–95 mmHg), who received one tablet/day containing 300 mg of Taurisolo^®^ plus 200 mg L-arginine. Treatment Group 2 included subjects with grade 2 hypertension (SBP 151–170 mmHg, DBP 96–105 mmHg), who received two tablets/day of the same formulation. Control Group 1 comprised subjects with grade 1 hypertension receiving one tablet/day of placebo (maltodextrins), while Control Group 2 comprised subjects with grade 2 hypertension receiving two tablets/day of placebo. The interventions did not require specific medical procedures, training, or expertise, and no clinical procedures beyond routine practice were foreseen.

During the screening visit (V1, Day 1), participants underwent blood pressure (BP) measurement and administration of the SF-12 questionnaire to assess eligibility. Following enrollment, all subjects were scheduled for repeated assessments at predefined time points: screening (V1, Day 1), baseline/start of treatment (T0, V2, Day 28), 4 weeks of treatment (T1, V3, Day 56), 8 weeks of treatment (T2, V4, Day 84), 12 weeks of treatment (T3, V5, Day 112), and at the end of the follow-up period (T4). At each of these visits, BP was measured. The SF-12 questionnaire was administered at screening (V1), baseline (T0), and after 12 weeks of treatment (T3), under the supervision of the study physician. In addition, blood and urine samples were collected at baseline (T0) and at 12 weeks (T3) for biomarker analysis (Table 1).

To minimize placebo-related bias, a 4-week run-in period was implemented prior to treatment initiation. During this phase, all enrolled participants received placebo tablets. At the end of the run-in, the SF-12 questionnaire was re-administered, and results were compared with those obtained at screening. Subjects demonstrating marked sensitivity to placebo were excluded from the study.

To evaluate potential carry-over effects of the investigational nutraceutical formulation (Taurisolo^®^ plus L-arginine), a 4-week follow-up period was included after completion of the 12-week treatment phase. During follow-up, no treatment was administered; at the end of this period, participants underwent BP measurement and completed the SF-12 questionnaire.

Blood pressure was measured at each study visit by trained study personnel using a validated automated oscillometric device with an appropriately sized cuff. Participants were seated and rested for at least 5 min before measurement; three consecutive readings were taken at 1 min intervals on the same arm and the mean of the last two readings was recorded. To reduce white-coat and situational effects, measurements were performed in a quiet room, by the same staff when possible, and participants were asked to avoid caffeine, smoking, and vigorous exercise for at least 30 min prior to assessment.

### 2.2. Outcome Measures

The primary objective of this study is to evaluate the effects of a nutraceutical formulation containing Taurisolo^®^ and L-arginine on blood pressure management. The intervention is intended as a first aid for a non-elevated blood pressure or as an adjunct capable of enhancing the effects of existing commercial products.

The primary endpoint is the change in systolic blood pressure (SBP) and diastolic blood pressure (DBP) over a 12-week treatment period. The therapeutic target was defined as normalization of SBP and DBP values, or at minimum, a reduction of ≥10 mmHg in SBP and ≥5 mmHg in DBP compared with baseline.

Secondary endpoints included evaluation of fasting plasma glucose (FG), total cholesterol (TC), low-density lipoprotein cholesterol (LDL-C), high-density lipoprotein cholesterol (HDL-C), triglycerides (TG), aspartate aminotransferase (AST), and alanine aminotransferase (ALT). Renal function was assessed through microalbuminuria and glomerular filtration rate (GFR). This latter was calculated based on the application of the CKD-EPI equation, which estimates GFR based on serum creatinine, sex, race, and age [34]. Plasma FG, TC, TG, HDL-C, LDL-C, AST and ALT levels were evaluated using commercially biochemical assay kits (Bionova s.r.l., Naples, Italy).

Participants also completed the Short Form Health Survey (SF-12), a validated measure of health-related quality of life derived from the original 36-item SF-36, developed in the 1980s and later condensed into a 12-item format [35]. The SF-12 consists of multiple-choice items whose responses are combined into summary scales covering eight core domains: Physical Activity, Role Physical, Bodily Pain, General Health, Vitality, Social Functioning, Role Emotional, and Mental Health. Together, these domains provide a concise yet comprehensive assessment of both physical and psychological well-being.

### 2.3. Interventions

Participants are randomly assigned to one of four groups. (i) Individuals with grade 1 hypertension (systolic blood pressure 130–150 mmHg, diastolic 90–95 mmHg) receive either one tablet per day containing 300 mg of grape pomace extract plus 200 mg of L-arginine, or (ii) one tablet per day of placebo consisting of maltodextrin. (iii) Individuals with grade 2 hypertension (systolic 151–170 mmHg, diastolic 96–105 mmHg) receive either two tablets per day of the same active formulation, or (iiii) two tablets per day of placebo.

Randomization is performed using a computer-generated randomization sequence and participants will be randomly assigned to one of the four groups through simple randomization with a 1:1:1:1 allocation ratio. The study is conducted under a double-blind design, ensuring that neither participants nor study staff are aware of treatment assignments.

The randomization list will remain concealed to protect the allocation sequence until assignment and will be securely stored in a locked location at the experimental center. The generation of the allocation sequence and the randomization list will be separated through the use of sealed envelopes. These envelopes will be prepared by an individual not involved in the clinical trial, rendered opaque, sealed, stapled, and sequentially numbered according to the randomization list, and subsequently stored in a locked cabinet. The investigator responsible for enrolling participants and administering treatments will open the next envelope in sequence, thereby remaining blinded to the allocation list.

Taurisolo^®^ was derived from Aglianico grapes harvested in autumn 2024. Large-scale production of Taurisolo^®^ was carried out by MBMed (Turin, Italy).

Both treatments and placebo were prepared by “La Sorgente del Benessere” Company (Fiuggi, Italy). Periodic and standardized telephone interviews conducted by qualified personnel were also used to verify and promote adherence to the study protocol. All treatments are provided free of charge, and the intervention period lasts 12 weeks, during which participants take the assigned treatment daily. Blinding was maintained by ensuring that the formulations (both active and placebo) were identical in appearance, packaging, and labelling. Neither the participants nor the investigators were aware of the group assignments until the study’s conclusion.

### 2.4. Statistical Analysis

Statistical analysis was performed using GraphPad Prism (version 8.4.3). Quantitative variables were expressed as mean, median, standard deviation, and interquartile range, while qualitative variables were reported as absolute and relative frequencies.

Blood pressure values were compared across groups and time points according to data distribution. A two-factor repeated measures ANOVA were applied to assess differences between groups, across time, and their interaction followed by Tukey’s multiple comparisons post hoc test, while the results of the SF-12 questionnaire were analysed using the multiple *t*-tests with the Holm–Sidak correction (PRISM software package, Version 8, GraphPad Software Inc., San Diego, CA, USA). A *p*-value of <0.05 was considered statistically significant.

Sample size was estimated assuming an effect size of 0.30 (Cohen), α = 0.05, 80% power, three repeated measures per participant, and an intra-subject correlation of 0.60. Based on these parameters, 74 participants per group are required, for a total of 296 subjects.

## 3. Results

### 3.1. Clinical Parameters at Baseline and After 3 Months

Table 2 shows the baseline (T0) and 3-month (T3) values for various clinical parameters for the four study groups. At baseline, there were no significant differences between the groups in terms of key metabolic indicators. These included measures of fasting blood sugar levels, lipid profile (i.e., total cholesterol, HDL-Cholesterol, LDL-Cholesterol, and triglyceride concentrations). Moreover, liver enzyme levels, which provide insight into liver function, did not show significant changes across any of the study groups.

### 3.2. Blood Pressure Changes at Baseline, 3-Month, and Follow-Up Periods

Table 3 summarizes the BP values across the four study groups, reporting measurements taken at baseline (T0), after 3 months of intervention (T3), and during a follow-up period. As regards the SBP levels, the treatment groups for both Stage 1 and Stage 2 hypertension had similar systolic blood pressure levels to the respective control groups at baseline. However, after 3 months of treatment, significant reductions were observed in both the treated groups. Specifically, the stage 1 treatment group (H1-TRT) exhibited a mean decrease of 13.5 mmHg (from 141.1 ± 6.6 mmHg at T0 to 127.6 ± 6.1 mmHg at T3, *p* < 0.0001), a result that was highly statistically significant when compared to both baseline and the control group (H1-CTR), which showed a slight increase of 1.6 mmHg. Similarly, in Stage 2 hypertension, the treatment group (H2-TRT) demonstrated a more substantial reduction of 17.4 mmHg (from 159.9 ± 6.3 mmHg at T0 to 142.5 ± 9.3 mmHg at T3, *p* < 0.0001), again showing a significant difference compared to the baseline and the control group (H2-CTR), whose systolic pressure remained unchanged (159.9 ± 6.1 mmHg at T3, with a minimal decrease of only 1.0 mmHg). During the follow-up phase, the reductions in SPB were generally sustained, with the H1-TRT group maintaining a mean systolic pressure of 131.4 ± 7.1 mmHg, and the H2-TRT group showing a follow-up measurement of 143.6 ± 9.7 mmHg.

Similar trends were observed in DBP measurements. For Stage 1 hypertension, the treatment group (H1-TRT) showed a significant reduction of 9.9 mmHg (from 92.6 ± 1.6 mmHg at baseline to 82.7 ± 1.7 mmHg at 3 months, *p* < 0.0001), and this effect was still evident at follow-up, with a mean diastolic value of 82.2 ± 1.6 mmHg. The control group (H1-CTR) did not show significant changes, indicating no effect in the absence of treatment. For Stage 2 hypertension, a more pronounced reduction of 18.5 mmHg (from 100.0 ± 2.8 mmHg at baseline to 81.5 ± 7.3 mmHg at 3 months, *p* < 0.0001) was observed in the H2-TRT group. This significant drop was maintained at the follow-up, where the mean diastolic pressure was 83.2 ± 7.2 mmHg. In contrast, the H2-CTR group saw only a small increase in diastolic pressure (+0.4 mmHg).

### 3.3. Markers of Kidney Function at Baseline and After 3 Months

Renal function parameters remained stable across the study groups between baseline (T0) and 3 months (T3) (Table 4). Microalbuminuria values showed no significant changes in either stage 1 or stage 2 cohorts. In stage 1, the treatment group (H1-TRT) increased slightly from 7.7 ± 10.5 mg/L at baseline to 8.3 ± 11.5 mg/L at 3 months, while the control group (H1-CTR) rose from 6.8 ± 8.9 to 10.8 ± 15.3 mg/L. In stage 2, H2-TRT values were 15.5 ± 11.9 at baseline and 16.5 ± 14.2 mg/L at 3 months, whereas H2-CTR showed a modest decrease from 15.8 ± 14.7 to 14.8 ± 13.1 mg/L.

Glomerular filtration rate (eGFR) also remained within comparable ranges across groups. In stage 1, H1-TRT increased from 70.7 ± 22.4 to 73.3 ± 19.4 mL/min/1.73 m^2^, while H1-CTR rose from 72.3 ± 20.5 to 74.3 ± 23.4. In stage 2, H2-TRT showed a slight reduction from 73.0 ± 22.6 to 69.3 ± 16.7, whereas H2-CTR remained stable (75.2 ± 18.1 to 74.3 ± 18.1).

Overall, no statistically significant differences were observed between treatment and control groups, confirming that supplementation with grape pomace extract (Taurisolo^®^) and L-arginine did not adversely affect renal function.

### 3.4. Health-Related Quality of Life

**Figure 3:**
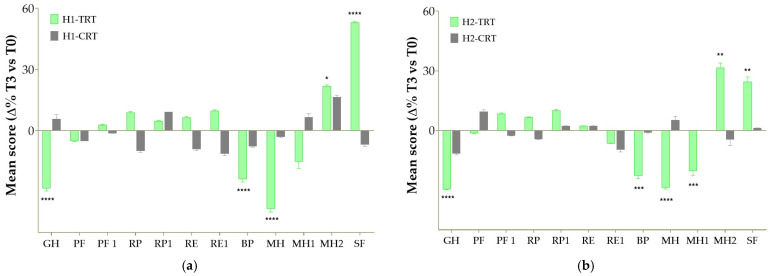
Percentage of variation in SF-12 domain scores after 3 months of intervention in the study groups with (**a**) hypertension stage 1 (H1) and (**b**) hypertension stage 2 (H2). Values are means ± standard deviation. (**a**) Data were analyzed with Multiple *t*-Tests. * *p* < 0.05 vs. T0 within the same group; **** *p* < 0.0001 vs. T0 within the same group. (**b**) Data were analyzed with Multiple *t*-Tests. ** *p* < 0.01 vs. T0 within the same group; *** *p* < 0.001 vs. T0 within the same group; **** *p* < 0.0001 vs. T0 within the same group. Abbreviations can be found in “Abbreviations” Section.

Health-related quality of life was assessed using the SF-12 questionnaire, a validated tool composed of 12 items that examine both physical and mental health domains. The scale covers key dimensions of health status, including physical functioning, role limitations, pain, general health, vitality, social functioning, and emotional well-being. In the context of this study, the SF-12 was employed to evaluate whether the formulation of Taurisolo^®^ and L-arginine could lead to improvements in participants’ perceived health status and allowed for evaluation of the intervention’s impact over the 3-month study period. In both hypertension stages, patients receiving the nutraceutical formulation reported a marked improvement in self-perceived general health compared with baseline (Figure 3). In H1-TRT, the General Health (GH) item score decreased by 28.4% (*p* < 0.0001 vs. T0), indicating a substantial shift toward better self-rated health, whereas the corresponding placebo group (H1-CTR) showed only a small, non-significant 5.8% increase (ns). Similarly, in the H2-TRT group, GH scores improved by 29.6% (*p* < 0.0001 vs. T0), while the H2-CTR group exhibited a more modest, non-significant variation. Improvements were also observed for Bodily Pain (BP) where pain-related interference improved by 23.8% (*p* < 0.0001) in the H1-TRT group and by 22.8% (*p* < 0.0001) in the H2-TRT group, indicating that perceived pain interfered less with usual activities after 12 weeks of active treatment. The intervention also produced notable benefits in mental well-being. Feeling of calmness and emotional balance (MH domain) improved by 38.3% in stage 1 hypertension and by 28.7% in stage 2 hypertension (*p* < 0.0001 for both). Vitality (MH1) followed a similar trajectory, although reaching statistical significance only in the H2-TRT group (+20.3%, *p* < 0.001). Placebo groups again showed no meaningful change. Regarding mood (MH2, “feeling downhearted and blue”), the nutraceutical formulation produced clear improvements in both stages of hypertension (+ 21.9%, *p* < 0.05 and + 31.5%, *p* < 0.01 for H1-TRT and H2-TRT groups, respectively). Taken together, these results show that the nutraceutical combination exerts a consistent positive impact on multiple dimensions of mental health (i.e., calmness, vitality, and mood) in both stage 1 and stage 2 hypertensive patients, whereas placebo does not. Nonetheless, social functioning (SF) markedly improved in patients receiving active treatment. In the H1-TRT group, SF scores increased by 53.2% (*p* < 0.0001), with the H2-TRT group also experiencing a significant improvement (+ 24.4%, *p* = 0.01).

## 4. Discussion

Most hypertensive patients require pharmacological therapy alongside lifestyle changes to achieve adequate blood pressure control. Recommended drug classes—ACE inhibitors, ARBs, beta blockers, alpha blockers, calcium channel blockers, and thiazide/thiazide-like diuretics—are supported by strong evidence from clinical trials and meta-analyses showing reduced cardiovascular events, with benefits largely attributable to blood pressure reduction [13,14]. However, these agents are frequently associated with adverse effects, including cough (ACE inhibitors), dizziness (ARBs), rashes and edema (CCBs), gastrointestinal disturbances (diuretics), bradycardia and sleep issues (β-blockers), and postural hypotension or syncope (α-blockers) [15].

In recent years, there has been a notable shift towards targeting sGC-cGMP pathway to regulate blood pressure and enhance cardiovascular health, especially in the context of hypertension and heart failure [36]. The significance of cGMP as a key regulator in maintaining the functional homeostasis of the cardiovascular system has been well established. Various therapeutic strategies aim to boost cGMP levels, either by donating NO, inhibiting its degradation via phosphodiesterase (PDE) inhibitors, or through the use of ACE inhibitors and aldosterone antagonists [37]. More recently, direct sGC stimulators, such as Vericiguat, have been introduced, which work by improving the activity of sGC and sensitizing it to endogenous NO, thereby promoting cGMP production and restoring functional cardiovascular regulation [38].

The combination of grape pomace extract (Taurisolo^®^) and L-arginine, as tested in this study, similarly targets this pathway by providing an additive source of L-arginine, the precursor to NO, which in turn stimulates the production of cGMP through sGC activation. By increasing NO bioavailability, this nutraceutical formulation directly influences the sGC-cGMP signaling cascade, which is crucial for blood pressure regulation. Grape pomace is rich in polyphenols [32], which have been shown to improve endothelial function, reduce oxidative stress, and modulate vascular tone [30,39,40]. Indeed, as already observed in previous in vitro and ex vivo studies [27], there is a synergistic action between L-arginine and Taurisolo^®^: the amino acid serves as the endogenous substrate of eNOS [41], thereby enhancing vasodilation and contributing to blood pressure reduction [42], whereas Taurisolo^®^ has a double function, acting as a potent antioxidant capable of counteracting TMAO-mediated inhibition of the enzyme (indirect action) and, by preventing oxidation of the heme Fe^2+^ atom, preserving sGC activity and subsequent cGMP production to maintain vascular homeostasis (direct action). Clinical investigations have confirmed the protective vascular effects of Taurisolo^®^ [43]. In a randomized, double-blind, placebo-controlled trial conducted in healthy volunteers, Taurisolo^®^ supplementation significantly improved endothelial function, as demonstrated by enhanced flow-mediated dilation (FMD) and increased NO bioavailability. These improvements were accompanied by a reduction in circulating biomarkers of oxidative stress, supporting the compound’s antioxidant activity observed in vitro and ex vivo. Importantly, Taurisolo^®^ was well tolerated, with no major adverse effects reported, underscoring its safety profile [30].

The observed reductions in systolic and diastolic blood pressure in both grade 1 and grade 2 hypertensive patients are likely a result of this mechanism, highlighting the potential of such nutraceuticals as adjunct therapies. This approach differs from traditional antihypertensive drugs, which often target broader physiological systems and are associated with various side effects. Moreover, while Vericiguat represents a pharmaceutical approach to stimulate sGC in heart failure directly, the natural combination used in this study provides a non-pharmacological option that could complement existing therapies. Importantly, the reductions were sustained during the follow-up period, indicating a potential long-term benefit of the intervention.

The treatment did not adversely affect renal function as shown in the kidney function table (Table 4), markers such as estimated glomerular filtration rate (eGFR) and microalbuminuria remained stable across all groups throughout the study period. No significant differences were observed between treatment and control groups at baseline, 3 months, or follow-up. These findings support the renal safety of the intervention and reinforce its suitability for long-term use in hypertensive populations, including those at risk of renal impairment.

The observed improvements in quality of life further support the clinical utility of the treatment. These data suggest a robust and clinically meaningful reduction in perceived pain and a clear improvement in global health status restricted to the actively treated patients. Considering stage 1 and stage 2 patients together, a coherent pattern emerges: the combined Taurisolo^®^ + L-arginine formulation produces substantial, statistically significant improvements in key SF-12 domains that reflect overall health perception (GH), emotional well-being and vitality (MH, MH1, MH2), and social participation (SF). The overall SF-12 profile supports a clinically relevant enhancement of health-related quality of life in hypertensive patients treated with the nutraceutical combination, over and above the changes observed with placebo.

## 5. Conclusions

In conclusion, supplementation with Taurisolo^®^ and L-arginine was associated with significant reductions in blood pressure and improvements in quality of life in patients with grade 1 and grade 2 hypertension. These findings support the potential role of nutraceuticals as adjunctive strategies in hypertension management, particularly for individuals seeking non-pharmacological approaches or those at risk of polypharmacy. The intervention proved to be safe and well tolerated, with no negative effects on renal function, and produced meaningful benefits across several quality-of-life domains, including general health perception, emotional well-being, vitality, and social participation, suggesting a positive impact on both physiological parameters and patient-reported outcomes.

Overall, these data indicate that the Taurisolo^®^ + L-arginine combination may represent a valuable complementary nutraceutical option alongside conventional antihypertensive therapies, contributing to improved blood pressure control and overall patient well-being. Future studies, including mechanistic investigations and larger multicenter trials of longer duration, will be necessary to confirm these findings and further elucidate the therapeutic potential of this formulation in the treatment of hypertension.

## 6. Patents

The following patent application related to the nutraceutical formulation investigated in this study has been filed and is currently pending: Patent Application No. 102024000007549, filed in 2024. The application concerns the formulation and composition of the dietary supplement used in this trial. Ownership of the application resides with NGN Healthcare. Authors Gian Carlo Tenore and Ettore Novellino are listed as inventors on this application.

## Figures and Tables

**Table 1:** Study outline.

Procedure	Screening Day 1 (V1)	End of Run-In Visit Day 28 (V2-T0)	Treatment Visit Day 56 (V3-T1)	Treatment Visit Day 84 (V4-T2)	Treatment Visit Day 112 (V5-T3)	End of Follow-Up Visit Day 140 (V6)
Informed consent and information sheet	√					
Eligibility assessment (inclusion/exclusion)	√					
Medical History	√					
Demographic data	√					
Blood pressure measurement	√	√	√	√	√	√
Placebo dispensation for run-in (amount for 4 weeks)	√					
Study treatment dispensation (amount for 12 weeks)		√	√	√		
SF-12 questionnaire administration	√	√			√	√
Blood and urine sample collection (biomarkers)		√			√	
Adverse events investigation			√	√	√	
Compliance assessment			√	√	√	
Concomitant medications recording	√	√	√	√	√	√

**Table 2:** Baseline and 3-month parameters in the four study groups.

	H1-TRT		H1-CRT		H2-TRT		H2-CRT	
	T0	T3	T0	T3	T0	T3	T0	T3
Male/Female								
Age (years)	45.8 ± 15.3	-	48.8 ± 18.5	-	55.5 ± 14.7	-	53.9 ± 12.1	-
FPG (mg/dL)	83.7 ± 10.3	84.0 ± 7.2	86.5 ± 10.5	87.8 ± 10.7	86.6 ± 11.1	86.5 ± 8.4	88.0 ± 11.8	88.1± 8.1
TC (mg/dL)	153.4 ± 28.9	160.4 ± 27.4	156.0 ± 27.2	155.2 ± 23.3	156.7 ± 27.4	153.1 ± 28.5	156.1 ± 25.3	150.9 ± 23.1
HDL-C (mg/dL)	80.3 ± 32.7	87.8 ± 28.4	80.9 ± 27.4	79.0 ± 24.5	79.1 ± 28.1	79.0 ± 29.1	78.0 ± 25.5	77.6 ± 24.8
LDL (mg/dL)	54.3 ± 15.5	53.2 ± 15.4	53.6 ± 13.1	57.3 ± 15.7	55.7 ± 16.2	51.4 ± 14.9	56.0 ± 12.2	51.5 ± 15.7
TG (mg/dL)	94.0 ± 37.5	97.2 ± 38.4	107.2 ± 39.4	94.5 ± 36.2	109.7 ± 38.5	114.0 ± 40.1	110.6 ± 38.3	109.0 ± 36.5
ALT (UI/L)	21.9 ± 10.0	21.0 ± 9.9	20.0 ± 10.9	19.5 ± 10.7	21.9 ± 10.4	19.8 ± 8.9	17.5 ± 7.6	18.6 ± 8.3
AST (UI/L)	26.2 ± 13.5	24.0 ± 10.7	22.7 ± 12.6	27.6 ± 11.3	30.3 ± 12.7	22.0 ± 9.9	24.7 ± 12.4	24.0 ± 12.5

Values are means ± standard deviation. Data were analyzed with One-way ANOVA followed by Tukey’s multiple comparisons post hoc test.

**Table 3:** Baseline, 3-month and follow-up blood pressure parameters in the four study groups.

	Systolic Blood Pressure (mmHg)			
	T0	T3	∆ T3 vs. T0	Follow-Up
H1-TRT	141.1 ± 6.6	127.6 ± 6.1 ^a4,b4^	−13.5	131.4 ± 7.1 ^a4,b4^
H1-CTR	138.5 ± 5.8	140.1 ± 6.5	+1.6	138.9 ± 6.5
H2-TRT	159.9 ± 6.3	142.5 ± 9.3 ^a4,b4^	−17.4	143.6 ± 9.7 ^a4,b4^
H2-CTR	160.6 ± 4.7	159.9 ± 6.1	−1.0	159.9 ± 5.7
	Diastolic Blood Pressure (mmHg)			
	T0	T3	∆ T3 vs. T0	Follow-Up
H1-TRT	92.6 ± 1.6	82.7 ± 1.7 ^a4,b4^	−9.9	82.2 ± 1.6 ^a4,b4^
H1-CTR	92.3 ± 1.8	92.2 ± 1.9	−0.1	92.3 ± 1.6
H2-TRT	100.0 ± 2.8	81.5 ± 7.3	−18.5	83.2 ± 7.2
H2-CTR	100.6 ± 2.6	100.6 ± 2.9	+ 0.4	100.2 ± 2.8

Values are means ± standard deviation. Data were analyzed with One-way ANOVA followed by Tukey’s multiple comparisons post hoc test; ^a4^ < 0.0001 vs. T0 within the same group; ^b4^ < 0.0001 significantly different vs. the Control group with the same stage of hypertension at the same time point.

**Table 4:** Baseline, 3-month markers of kidney function in the four study groups.

	Microalbuminuria (mg/L)	
	T0	T3
H1-TRT	7.7 ± 10.5	8.3 ± 11.5
H1-CTR	6.8 ± 8.9	10.8 ± 15.3
H2-TRT	15.5 ± 11.9	16.5 ± 14.2
H2-CTR	15.8 ± 14.7	14.8 ± 13.1
	Glomerular Filtration Rate (mL/min/1.73 m^2^)	
	T0	T3
H1-TRT	70.7 ± 22.4	73.3 ± 19.4
H1-CTR	72.3 ± 20.5	74.3 ± 23.4
H2-TRT	73.0 ± 22.6	69.3 ± 16.7
H2-CTR	75.2 ± 18.1	74.3 ± 18.1

Values are means ± standard deviation.

## Data Availability

The original contributions presented in this study are included in the article/Supplementary Material. Further inquiries can be directed to the corresponding author.

## Supplementary Materials

The following supporting information can be downloaded at: https://www.mdpi.com/article/10.3390/antiox15030329/s1, Table S1: Average values with Standard Deviation (±SD) of Systolic Blood Pressure (SBP), Diastolic Blood Pressure (DBP), Glycemia (GLY), Total Cholesterol (TC), Low Density Lipoprotein (LDL), High Density Lipoprotein (HDL), Microalbuminuria (MA) and Body Mass Index (BMI) of male and female patients of H1-TRT group at T0 and T3; Table S2: Δ% values indicate the percentage change from baseline (T0) to the three months assessment (T3) for all clinical parameters in male and female subjects enrolled in the H1 TRT group; Table S3: Average values with Standard Deviation (±SD) of Systolic Blood Pressure (SBP), Diastolic Blood Pressure (DBP), Glycemia (GLY), Total Cholesterol (TC), Low Density Lipoprotein (LDL), High Density Lipoprotein (HDL), Microalbuminuria (MA) and Body Mass Index (BMI) of male and female patients of H1-CTR group at T0 and T3; Table S4: Δ% values indicate the percentage change from baseline (T0) to the three month assessment (T3) for all clinical parameters in male and female subjects enrolled in the H1 CTR group; Table S5: Average values with Standard Deviation (±SD) of Systolic Blood Pressure (SBP), Diastolic Blood Pressure (DBP), Glycemia (GLY), Total Cholesterol (TC), Low Density Lipoprotein (LDL), High Density Lipoprotein (HDL), Microalbuminuria (MA) and Body Mass Index (BMI) of male and female patients of H2-TRT group at T0 and T3; Table S6: Δ% values indicate the percentage change from baseline (T0) to the three months assessment (T3) for all clinical parameters in male and female subjects enrolled in the H2 TRT group; Table S7: Average values with Standard Deviation (±SD) of Systolic Blood Pressure (SBP), Diastolic Blood Pressure (DBP), Glycemia (GLY), Total Cholesterol (TC), Low Density Lipoprotein (LDL), High Density Lipoprotein (HDL), Microalbuminuria (MA) and Body Mass Index (BMI) of male and female patients of H2-CTR group at T0 and T3; Table S8: Δ% values indicate the percentage change from baseline (T0) to the three month assessment (T3) for all clinical parameters in male and female subjects enrolled in the H2 CTR group.

## Abbreviations

The following abbreviations are used in this manuscript:

AF	Atrial Fibrillation
ALT	Alanine Aminotransferase
AST	Aspartate Aminotransferase
BP	Blood Pressure
cGMP	Cyclic Guanosine Monophosphate
CCB	Calcium Channel Blocker
CKD-EPI	Chronic Kidney Disease Epidemiology Collaboration
CONSORT	Consolidated Standards of Reporting Trials
DALY	Disability-Adjusted Life Year
DBP	Diastolic Blood Pressure
eNOS	Endothelial Nitric Oxide Synthase
FG/FPG	Fasting Plasma Glucose
GFR	Glomerular Filtration Rate
GT/γ-GT	Gamma-Glutamyl Transferase
Hb	Hemoglobin
HDL-C	High-Density Lipoprotein Cholesterol
HNOX	Heme Nitric Oxide/Oxygen Binding Domain
H1-CTR	Hypertension Grade 1—Control
H1-TRT	Hypertension Grade 1—Treatment
H2-CTR	Hypertension Grade 2—Control
H2-TRT	Hypertension Grade 2—Treatment
iNOS	Inducible Nitric Oxide Synthase
ISRCTN	International Standard Randomised Controlled Trial Number
LDL-C/LDL	Low-Density Lipoprotein Cholesterol
NO	Nitric Oxide
NOS	Nitric Oxide Synthase
nNOS	Neuronal Nitric Oxide Synthase
PKG	Protein Kinase G
RE	Role Emotional (SF-12 domain)
RP	Role Physical (SF-12 domain)
SBP	Systolic Blood Pressure
sGC	Soluble Guanylate Cyclase
SF	Social Functioning (SF-12 domain)
SF-12	Short Form-12 Health Survey
SF-36	Short Form-36 Health Survey
SNP	Sodium Nitroprusside
TC	Total Cholesterol
TG	Triglycerides
TMAO	Trimethylamine N-Oxide
TRT	Treatment
V1, V2, …	Visit numbers in the clinical protocol
YLDs	Years Lived with Disability
YLLs	Years of Life Lost

## References

[1] Johnson H.M., Shimbo D., Abdalla M., Altieri M.M., Bress A.P., Carter J., Commodore-Mensah Y., Egan B., Melnyk B.M., Ogunniyi M.O. (2025). 2025 AHA/ACC/AANP/AAPA/ABC/ACCP/ACPM/AGS/AMA/ASPC/NMA/PCNA/SGIM Guideline for the Prevention, Detection, Evaluation and Management of High Blood Pressure in Adults: A Report of the American College of Cardiology/American Heart Association Joint Committee on Clinical Practice Guidelines. Hypertension.

[2] Mills K.T., Stefanescu A., He J. (2020). The Global Epidemiology of Hypertension. Nat. Rev. Nephrol..

[3] Yano Y., Stamler J., Garside D.B., Daviglus M.L., Franklin S.S., Carnethon M.R., Liu K., Greenland P., Lloyd-Jones D.M. (2015). Isolated Systolic Hypertension in Young and Middle-Aged Adults and 31-Year Risk for Cardiovascular Mortality: The Chicago Heart Association Detection Project in Industry Study. J. Am. Coll. Cardiol..

[4] Borghi C., Dormi A., L’Italien G., Lapuerta P., Franklin S.S., Collatina S., Gaddi A. (2003). The Relationship Between Systolic Blood Pressure and Cardiovascular Risk—Results of the Brisighella Heart Study. J. Clin. Hypertens..

[5] Lionakis N., Mendrinos D., Sanidas E., Favatas G., Georgopoulou M. (2012). Hypertension in the Elderly. World J. Cardiol..

[6] Basile J.N. (2002). Systolic Blood Pressure. BMJ.

[7] Ettehad D., Emdin C.A., Kiran A., Anderson S.G., Callender T., Emberson J., Chalmers J., Rodgers A., Rahimi K. (2016). Blood Pressure Lowering for Prevention of Cardiovascular Disease and Death: A Systematic Review and Meta-Analysis. Lancet.

[8] World Health Organization (2020). WHO Methods and Data Sources for Global Burden of Disease Estimates 2000–2019.

[9] Forouzanfar M.H., Afshin A., Alexander L.T., Biryukov S., Brauer M., Cercy K., Charlson F.J., Cohen A.J., Dandona L., Estep K. (2016). Global, Regional, and National Comparative Risk Assessment of 79 Behavioural, Environmental and Occupational, and Metabolic Risks or Clusters of Risks, 1990–2015: A Systematic Analysis for the Global Burden of Disease Study 2015. Lancet.

[10] Chong B., Jayabaskaran J., Jauhari S.M., Chan S.P., Goh R., Kueh M.T.W., Li H., Chin Y.H., Kong G., Anand V.V. (2025). Global Burden of Cardiovascular Diseases: Projections from 2025 to 2050. Eur. J. Prev. Cardiol..

[11] Verdecchia P., Angeli F., Reboldi G. (2018). Hypertension and Atrial Fibrillation: Doubts and Certainties from Basic and Clinical Studies. Circ. Res..

[12] Masenga S.K., Kirabo A. (2023). Hypertensive Heart Disease: Risk Factors, Complications and Mechanisms. Front. Cardiovasc. Med..

[13] World Health Organization Hypertension. https://www.who.int/news-room/fact-sheets/detail/hypertension.

[14] Zhu J., Chen N., Zhou M., Guo J., Zhu C., Zhou J., Ma M., He L. (2022). Calcium Channel Blockers versus Other Classes of Drugs for Hypertension. Cochrane Database Syst. Rev..

[15] Tedla Y.G., Bautista L.E. (2016). Drug Side Effect Symptoms and Adherence to Antihypertensive Medication. Am. J. Hypertens..

[16] Benson J., Britten N. (2003). Patients’ Views about Taking Antihypertensive Drugs: Questionnaire Study. BMJ.

[17] Vrijens B., Vincze G., Kristanto P., Urquhart J., Burnier M. (2008). Adherence to Prescribed Antihypertensive Drug Treatments: Longitudinal Study of Electronically Compiled Dosing Histories. BMJ.

[18] Stasch J.P., Pacher P., Evgenov O.V. (2011). Soluble Guanylate Cyclase as an Emerging Therapeutic Target in Cardiopulmonary Disease. Circulation.

[19] Belenkov Y.N., Kozhevnikova M.V. (2023). Soluble Guanylate Cyclase: Restoration of the NO–SGC–CGMP Signaling Pathway Activity. A New Opportunity in the Treatment of Heart Failure. Kardiologiya.

[20] Murad F. (2006). Nitric Oxide and Cyclic GMP in Cell Signaling and Drug Development. N. Engl. J. Med..

[21] Montfort W.R., Wales J.A., Weichsel A. (2017). Structure and Activation of Soluble Guanylyl Cyclase, the Nitric Oxide Sensor. Antioxid. Redox Signal..

[22] Dysregulation of Nitric Oxide/CGMP Pathway: A Pathological Cascade in Vascular Dysfunction|Request PDF. https://www.researchgate.net/publication/378677554_Dysregulation_of_Nitric_OxidecGMP_Pathway_A_Pathological_Cascade_in_Vascular_Dysfunction.

[23] Thoonen R., Sips P.Y., Bloch K.D., Buys E.S. (2013). Pathophysiology of Hypertension in the Absence of Nitric Oxide/Cyclic GMP Signaling. Curr. Hypertens. Rep..

[24] Fernhoff N.B., Derbyshire E.R., Underbakke E.S., Marletta M.A. (2012). Heme-Assisted S-Nitrosation Desensitizes Ferric Soluble Guanylate Cyclase to Nitric Oxide. J. Biol. Chem..

[25] Lubos E., Handy D.E., Loscalzo J. (2008). Role of Oxidative Stress and Nitric Oxide in Atherothrombosis. Front. Biosci..

[26] Kondapalli N., Katari V., Dalal K.K., Paruchuri S., Thodeti C.K. (2025). Microbiota in Gut-Heart Axis: Metabolites and Mechanisms in Cardiovascular Disease. Compr. Physiol..

[27] Martelli A., Abate F., Roggia M., Benedetti G., Caradonna E., Calderone V., Tenore G.C., Cosconati S., Novellino E., Stornaiuolo M. (2025). Trimethylamine N-Oxide (TMAO) Acts as Inhibitor of Endothelial Nitric Oxide Synthase (ENOS) and Hampers NO Production and Acetylcholine-Mediated Vasorelaxation in Rat Aortas. Antioxidants.

[28] Koress C., Swan K., Kadowitz P. (2016). Soluble Guanylate Cyclase Stimulators and Activators: Novel Therapies for Pulmonary Vascular Disease or a Different Method of Increasing CGMP?. Curr. Hypertens. Rep..

[29] Goszcz K., Duthie G.G., Stewart D., Leslie S.J., Megson I.L. (2017). Bioactive Polyphenols and Cardiovascular Disease: Chemical Antagonists, Pharmacological Agents or Xenobiotics That Drive an Adaptive Response?. Br. J. Pharmacol..

[30] Martelli A., Flori L., Gorica E., Piragine E., Saviano A., Annunziata G., Di Minno M.N.D., Ciampaglia R., Calcaterra I., Maione F. (2021). Vascular Effects of the Polyphenolic Nutraceutical Supplement Taurisolo®: Focus on the Protection of the Endothelial Function. Nutrients.

[31] Lapi D., Tenore G.C., Federighi G., Chiurazzi M., Nunziato S., Lonardo M.S., Stornaiuolo M., Colantuoni A., Novellino E., Scuri R. (2024). L-Arginine and Taurisolo® Effects on Brain Hypoperfusion–Reperfusion Damage in Hypertensive Rats. Int. J. Mol. Sci..

[32] Karastergiou A., Gancel A.L., Jourdes M., Teissedre P.L. (2024). Valorization of Grape Pomace: A Review of Phenolic Composition, Bioactivity, and Therapeutic Potential. Antioxidants.

[33] Calvert M., Blazeby J., Altman D.G., Revicki D.A., Moher D., Brundage M.D. (2013). Reporting of Patient-Reported Outcomes in Randomized Trials: The CONSORT PRO Extension. JAMA.

[34] Levey A.S., Stevens L.A., Schmid C.H., Zhang Y., Castro A.F., Feldman H.I., Kusek J.W., Eggers P., Van Lente F., Greene T. (2009). A New Equation to Estimate Glomerular Filtration Rate. Ann. Intern. Med..

[35] Huo T., Guo Y., Shenkman E., Muller K. (2018). Assessing the Reliability of the Short Form 12 (SF-12) Health Survey in Adults with Mental Health Conditions: A Report from the Wellness Incentive and Navigation (WIN) Study. Health Qual. Life Outcomes.

[36] Breitenstein S., Roessig L., Sandner P., Lewis K.S. (2017). Novel SGC Stimulators and SGC Activators for the Treatment of Heart Failure. Handb. Exp. Pharmacol..

[37] Greenberg B. (2016). Novel Therapies for Heart Failure—Where Do They Stand?. Circ. J..

[38] Follmann M., Ackerstaff J., Redlich G., Wunder F., Lang D., Kern A., Fey P., Griebenow N., Kroh W., Becker-Pelster E.M. (2017). Discovery of the Soluble Guanylate Cyclase Stimulator Vericiguat (BAY 1021189) for the Treatment of Chronic Heart Failure. J. Med. Chem..

[39] Annunziata G., Maisto M., Schisano C., Ciampaglia R., Narciso V., Tenore G.C., Novellino E., Annunziata G., Maisto M., Schisano C. (2019). Effects of Grape Pomace Polyphenolic Extract (Taurisolo®) in Reducing TMAO Serum Levels in Humans: Preliminary Results from a Randomized, Placebo-Controlled, Cross-Over Study. Nutrients.

[40] Lama S., Monda V., Rizzo M.R., Dacrema M., Maisto M., Annunziata G., Tenore G.C., Novellino E., Stiuso P. (2020). Cardioprotective Effects of Taurisolo® in Cardiomyoblast H9c2 Cells under High-Glucose and Trimethylamine N-Oxide Treatment via De Novo Sphingolipid Synthesis. Oxidative Med. Cell. Longev..

[41] Wu G., Meininger C.J., McNeal C.J., Bazer F.W., Rhoads J.M. (2021). Role of L-Arginine in Nitric Oxide Synthesis and Health in Humans. Adv. Exp. Med. Biol..

[42] Melik Z., Zaletel P., Virtic T., Cankar K. (2017). L-Arginine as Dietary Supplement for Improving Microvascular Function. Clin. Hemorheol. Microcirc..

[43] Amato B., Novellino E., Morlando D., Vanoli C., Vanoli E., Ferrara F., Difruscolo R., Goffredo V.M., Compagna R., Tenore G.C. (2024). Benefits of Taurisolo in Diabetic Patients with Peripheral Artery Disease. J. Cardiovasc. Dev. Dis..

