# Supplementary material for: A Nutraceutical Approach for Hypertension: Randomized Controlled Trial of Grape Pomace Extract and L-Arginine
Source: Antioxidants (Basel). 2026 Mar 5;15(3):329. doi: 10.3390/antiox15030329 (PMC13023866; doi:10.3390/antiox15030329)
Supplement: Supplementary file 1 [file antioxidants-15-00329-s001.zip › File_S2_H1-CTR.pdf]

	Sex	Age	T0 PAS	T0 PAD	T0 GLY	T0 TC	T0 LDL	T0 HDL	T0 MA	T0 BMI	T0 GFR	T3 PAS	T3 PAD	T3 GLY	T3 TC	T3 LDL	T3 HDL	T3 MA	T3 BMI	T3 GFR
**Average (±SD)**	M	56,3± 18,0	139,1 ± 6,1	92,9± 1,7	83,7± 9,7	169,5 ±23,9	91,7± 25,0	54,3± 12,3	8,6± 9,4	27,33± 1,18	79,90± 21,86	139,0 ± 6,3	91,9± 1,8	88,8± 12,1	148,2 ±24,5	71,2± 25,5	56,7± 13,4	12,1± 13,8	27,34± 1,17	82,63± 20,19
**Average (±SD)**	F	43,8± 17,6	138,1 ± 5,7	91,9± 1,8	88,3± 10,8	146,9 ±26,0	73,7± 27,2	53,2± 13,9	5,6± 8,7	26,49± 1,47	67,26± 18,34	140,8 ± 6,6	92,4± 1,9	87,2± 9,9	159,8 ±21,9	84,2± 23,2	57,7± 17,4	9,9± 16,6	26,61± 1,46	68,71± 24,26

Table S3. Average values with Standard Deviation (±SD) of Systolic Blood Pressure (SBP), Diastolic Blood Pressure (DBP), Glycemia (GLY), Total Cholesterol (TC), Low Density Lipoprotein (LDL), High Density Lipoprotein (HDL), Microalbuminuria (MA) and Body Mass Index (BMI) of male and female patients of H1-CTR group at T0 and T3.

Δ% T3 -T0										
	**Sex**	**PAS**	**PAD**	**GLY**	**TC**	**LDL**	**HDL**	**MA**	**BMI**	**GFR**
**Δ% Men**	M	-0,1	-1,1	+6,2	-12,6	-22,3	+4,5	+40,8	+0,04	-15,8
**Δ% Women**	F	+2,0	+0,6	-1,3	+8,8	+14,2	+8,4	+77,2	+0,45	-16,9
**Δ% (F-M)**		+2,1	+1,7	-7,5	+21,4	+36,5	+3,9	+36,4	+0,41	-1,1

Table S4. Δ% values indicate the percentage change from baseline (T0) to the three-month assessment (T3) for all clinical parameters in male and female subjects enrolled in the H1-CTR group.
