# Supplementary material for: A Nutraceutical Approach for Hypertension: Randomized Controlled Trial of Grape Pomace Extract and L-Arginine
Source: Antioxidants (Basel). 2026 Mar 5;15(3):329. doi: 10.3390/antiox15030329 (PMC13023866; doi:10.3390/antiox15030329)
Supplement: Supplementary file 1 [file antioxidants-15-00329-s001.zip › File_S3_H2-TRT.pdf]

		T0										T3								
	**Sex**	**Age**	**PAS**	**PAD**	**GLY**	**TC**	**LDL**	**HDL**	**MA**	**BMI**	**GFR**	**PAS**	**PAD**	**GLY**	**TC**	**LDL**	**HDL**	**MA**	**BMI**	**GFR**
**Average (±SD)**	M	54,4± 16,7	157,4 ± 5,9	99,0± 2,8	85,7± 9,6	156,7 ±29,4	80,0± 22,4	54,8± 17,6	16,3± 11,7	27,45± 0,83	86,25± 24,10	142,9 ± 7,5	81,5± 8,8	85,3± 6,7	159,8 ±30,4	85,1± 33,3	53,8± 16,3	16,3± 14,6	27,48± 0,84	77,22± 15,99
**Average (±SD)**	F	56,2± 13,7	161,6 ± 6,2	100,7± 2,6	87,3± 12,3	156,8 ±26,8	78,5± 32,0	56,3± 15,7	15,0± 12,4	26,93± 1,16	64,08± 16,91	142,3 ±10,5	81,6± 6,5	87,2± 9,4	148,7 ±27,2	74,9± 26,1	49,7± 14,2	16,7± 14,4	27,03± 1,17	64,09± 15,41

Table S5. Average values with Standard Deviation (±SD) of Systolic Blood Pressure (SBP), Diastolic Blood Pressure (DBP), Glycemia (GLY), Total Cholesterol (TC), Low Density Lipoprotein (LDL), High Density Lipoprotein (HDL), Microalbuminuria (MA) and Body Mass Index (BMI) of male and female patients of H2-TRT group at T0 and T3.

		Δ% T3 -T0								
	**Sex**	**PAS**	**PAD**	**GLY**	**TC**	**LDL**	**HDL**	**MA**	**BMI**	**GFR**
**Δ% Men**	M	-9,2	-17,7	-0,4	+2,0	+6,3	-1,8	+0,5	+0,11	-25,7
**Δ% Women**	F	-12,0	-19,0	-0,1	-5,2	-4,5	-11,6	+11,1	+0,37	-17,0
**Δ% (F-M)**		-2,8	-1,3	+0,3	-7,2	-10,8	-9,8	+10,6	+0,26	+8,7

Table S6. Δ% values indicate the percentage change from baseline (T0) to the three-month assessment (T3) for all clinical parameters in male and female subjects enrolled in the H2-TRT group.
